# Automatic 4D mitral valve segmentation from transesophageal echocardiography: a semi-supervised learning approach

**DOI:** 10.1007/s11517-024-03275-w

**Published:** 2025-01-11

**Authors:** Riccardo Munafò, Simone Saitta, Davide Tondi, Giacomo Ingallina, Paolo Denti, Francesco Maisano, Eustachio Agricola, Emiliano Votta

**Affiliations:** 1 Department of Electronics, Information and Bioengineering, Politecnico di Milano, Milan, Italy; 2 Department of Biomedical Engineering and Physics, Amsterdam UMC, Amsterdam, The Netherlands; 3 Unit of Cardiovascular Imaging, IRCCS San Raffaele Hospital, Milan, Italy; 4 Cardiac Surgery Department, IRCCS San Raffaele Hospital, Milan, Italy; 5 Vita-Salute San Raffaele University, Milan, Italy; 6 Informatics Institute, University of Amsterdam, Amsterdam, The Netherlands

**Keywords:** Three-dimensional transesophageal echocardiography, Mitral regurgitation, Automatic segmentation, Convolutional neural network, Semi-supervised learning, Mitral valve

## Abstract

**Abstract:**

Performing automatic and standardized 4D TEE segmentation and mitral valve analysis is challenging due to the limitations of echocardiography and the scarcity of manually annotated 4D images. This work proposes a semi-supervised training strategy using pseudo labelling for MV segmentation in 4D TEE; it employs a Teacher-Student framework to ensure reliable pseudo-label generation. 120 4D TEE recordings from 60 candidates for MV repair are used. The Teacher model, an ensemble of three convolutional neural networks, is trained on end-systole and end-diastole frames and is used to generate MV pseudo-segmentations on intermediate frames of the cardiac cycle. The pseudo-annotated frames augment the Student model’s training set, improving segmentation accuracy and temporal consistency. The Student outperforms individual Teachers, achieving a Dice score of 0.82, an average surface distance of 0.37 mm, and a 95% Hausdorff distance of 1.72 mm for MV leaflets. The Student model demonstrates reliable frame-by-frame MV segmentation, accurately capturing leaflet morphology and dynamics throughout the cardiac cycle, with a significant reduction in inference time compared to the ensemble. This approach greatly reduces manual annotation workload and ensures reliable, repeatable, and time-efficient MV analysis. Our method holds strong potential to enhance the precision and efficiency of MV diagnostics and treatment planning in clinical settings.

**Graphical abstract:**

**Figure:**
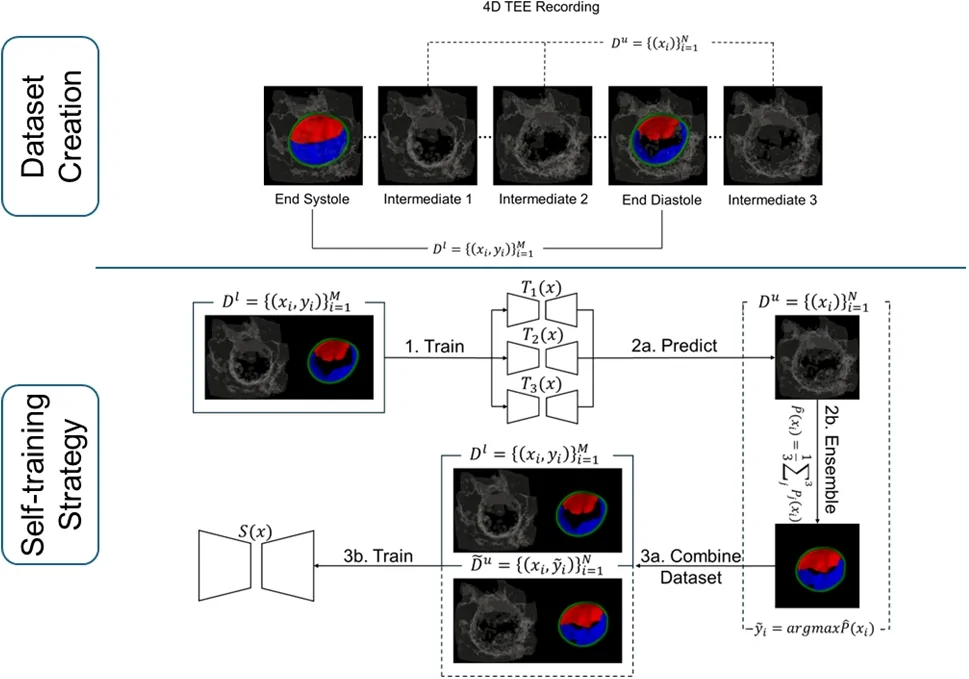

## Introduction

The mitral valve (MV) is a dynamic structure between the left ventricle and atrium, consisting of two leaflets anchored to a saddle-shaped annulus and connected to the myocardium by *chordae tendineae* that attach to the papillary muscles. In physiological MVs, during diastole the leaflets open wide and the annulus dilates to allow blood flow from the left atrium to the left ventricle. In systole, the leaflets close tightly and the annulus contracts to prevent backflow into the atrium, ensuring valve competence and optimal leaflet coaptation. Disruption of any MV component can lead to mitral regurgitation (MR) [1], the most widespread valvular pathology [2]. Echocardiography is the primary imaging modality for visualizing and assessing MV anatomy and function [3]. Specifically, transesophageal echocardiography (TEE) provides high-quality imaging of cardiac structures in 2D and 3D, enabling the dynamic evaluation of these structures in real-time. TEE is also adopted to monitor MV repair [7], including transcatheter edge-to-edge repair (TEER). Quantitative assessment of mitral annulus dynamics, leaflet mobility, and excursion is crucial in evaluating MR and characterizing its various etiologies [4, 5]. Furthermore, a dynamic quantification of MV morphology and kinematics can inform computational models, including finite element simulations that replicate patient-specific MV movement and mechanical properties [10, 11]. The quantification of the MV apparatus from 4D TEE, possibly distinguishing between its substructures, requires pixel-wise annotation. If not highly automated, this process is operator-dependent [12], as well as labor-intensive and time-consuming, often exceeding the time constraints of a standard examination, even for expert sonographers [13]. Automatic and standardized methods for 4D TEE annotation and MV morphology and motion quantification can reduce the sonographers’ workload and improve the analysis reliability and repeatability.

Automatic methods for MV segmentation and tracking from 4D TEE are challenging due to the well-known limitations of echocardiography, including motion artifacts, poor-image quality, and scarce availability of manually annotated 4D images. Convolutional neural networks (CNNs) have shown promising performance for supervised MV segmentation from echocardiography images. Costa et al. [20] implemented a 2D CNN for automatic MV leaflets segmentation from 2D transthoracic echocardiography (TTE) data. Carnahan et al. [21] and Aly et al. [22] proposed automatic 3D MV segmentation from 3D TEE images using a 3D Residual UNet and nnUNet, respectively. Chen et al. [23] proposed a two-step solution for 3D MV segmentation in systolic and diastolic configurations, initializing a nnUNet with pre-trained weights of a classifier for the MV open and closed states. Munafò et al. [33] developed a Multi-Decoder Residual UNet for multi-structure MV segmentation at end-systole (ES), enabling separate identification of the mitral annulus, anterior and posterior leaflets from 3D TEE images. Yet, none of the mentioned studies achieved the frame-by-frame quantification of MV anatomy in 4D TEE images. Indeed, so far this task has been performed either on 3D TEE imaging but with a focus only on annulus segmentation [25, 26] or tracking [27], or on annulus and leaflets but in 2D imaging, as in [24], who proposed an effective UNet with attention gates for segmenting 2D TTE images. Despite the advancements in neural network architectures, there is currently no method allowing for the full 4D MV segmentation within the short times required to meet the strict clinical demands of diagnostic echocardiographic screening, where large amounts of dataset must be analyzed, and intraprocedural guidance during percutaneous interventions, where real-time or almost real-time annotation of images is needed to provide operators with timely visual information. A significant bottleneck in the development of such a method is the labor-intensive manual annotation process needed to generate the extensive datasets required for the supervised training of CNNs.

**Fig. 1:**
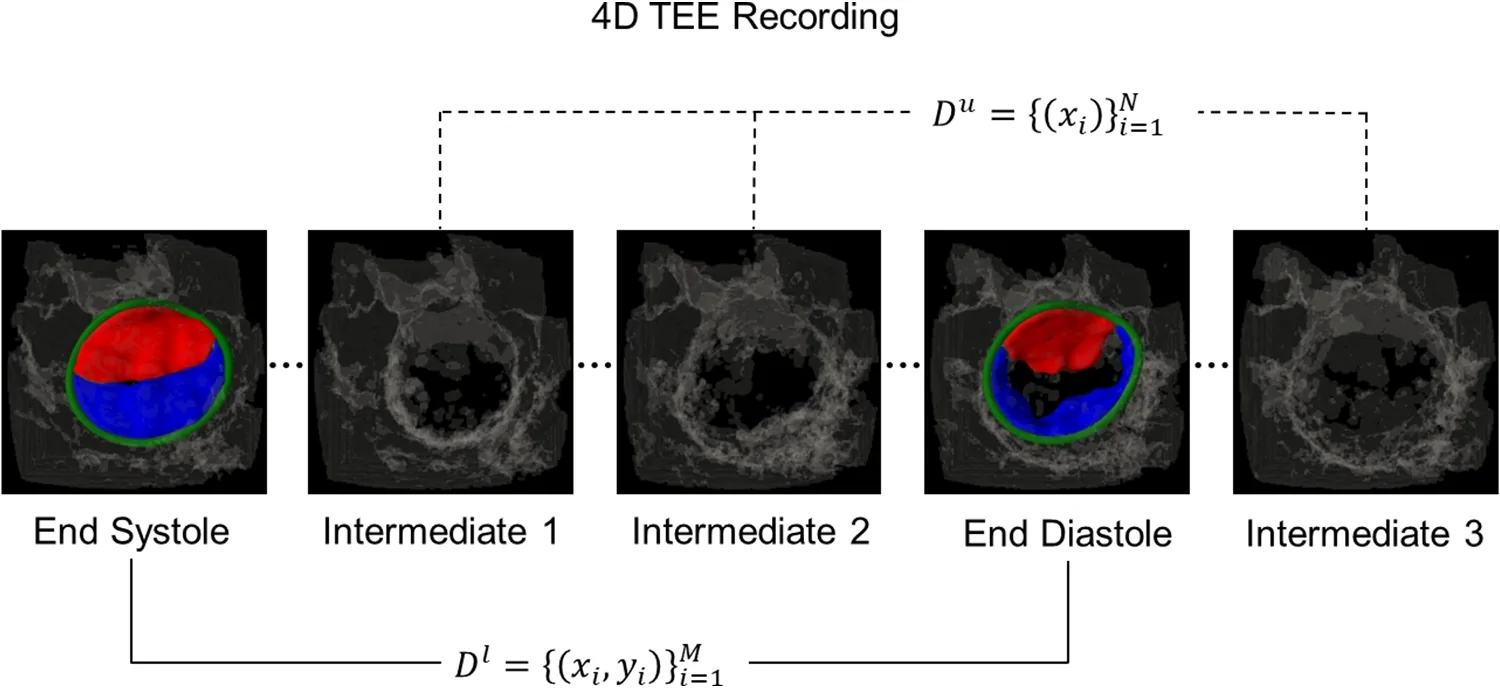
3D renderings of TEE frames extracted from each 4D TEE recording, where each frame consists of a 3D image volume. The ES and the ED frames, along with the corresponding 3D MV annotations, compose $D^{l}$. Three intermediate frames, belonging to the diastole and systole phases and picked to uniformly sample the cardiac cycle, compose $D^{u}$

This work addresses this gap by introducing a semi-supervised training strategy that reduces the need for manual annotations without compromising segmentation accuracy. Our approach leverages a semi-supervised training strategy using pseudo-labeling, which has never been applied in the context of 4D TEE MV segmentation. We use manual annotations of only the ES and end-diastole (ED) frames to achieve automatic segmentation of the MV throughout the cardiac cycle. Additionally, we propose an ensemble strategy to generate high-confidence pseudo-labels and evaluate the impact of specific data augmentation previously used in self-training strategies to ensure high consistency on the unlabeled dataset.

## Methods

### Dataset collection and annotation

Intraprocedural examinations were collected from 60 candidates for MV TEER using the MitraClip system (Abbott) at IRCCS San Raffaele Hospital (Milan, Italy). The cohort consisted of 21 females and 39 males, with ages ranging from 50 and 95 years. Acquisitions were performed with a Philips EPIQ CVx scanner and an X8-2T transducer (Philips, Andover, MA, USA). Image data were anonymized and exported in Cartesian format using QLab software (Philips, Andover, MA, USA). The study was approved by the local ethics committee. Midesophageal 4D TEE acquisitions, zoomed in on the MV, were selected from the collected examinations to construct the dataset. For some patients, multiple acquisitions were available and included in the dataset. Acquisitions with high shadowing or those that did not entirely capture the MV within the field of view of the ultrasound beam were excluded. The final dataset counted n=120 4D TEE recordings with a mean voxel spacing of 0.37 mm × 0.55 mm × 0.24 mm and a mean frame rate of 22 per cardiac cycle. This dataset is a slightly augmented version of the dataset used in our previous work [33], with a small increase in the number of patients considered. Additionally, manual 3D annotations of the MV at the ES frame from [33] were reused in this work and expanded to include MV annotations at ED. ES and ED were defined as the frame before the aortic valve closure and the frame before MV closure, respectively. ED volumes were annotated by three independent and experienced operators following the same protocol described in [33] and using 3D Slicer [43]. Multi-class annotations of the MV were produced by separately identifying the mitral annulus, the anterior leaflet, and the posterior leaflet.

### Semi-supervised segmentation strategy

Semi-supervised semantic segmentation aims to generalize using a combined set of voxel-wise labeled images $D^{l}=\{(x_{i}, y_{i})\}^{M}_{i=1}$ and unlabeled images $D^{u}=\{(x_{i})\}^{N}_{i=1}$, with *N* usually larger than *M*. In this work, the images are from *n* 4D TEE recordings of the MV covering a full cardiac cycle, where at each frame a volumetric image is available. $D^{l}$ consists of the 3D images at ES and ED frames extracted from each recording, along with the corresponding manually obtained 3D annotations of MV substructures. $D^{u}$ comprises the images at three intermediate frames evenly sampled over the remaining frames of the cardiac cycle (Fig. 1). Overall, $D^{l}$ and $D^{u}$ contain a total of M=2n and N=3n images, respectively. Both $D^{l}$ and $D^{u}$ are used complementary in a self-training learning strategy to train CNN models for multi-class segmentation of the MV.

### Self-training approach for MV segmentation

**Fig. 2:**
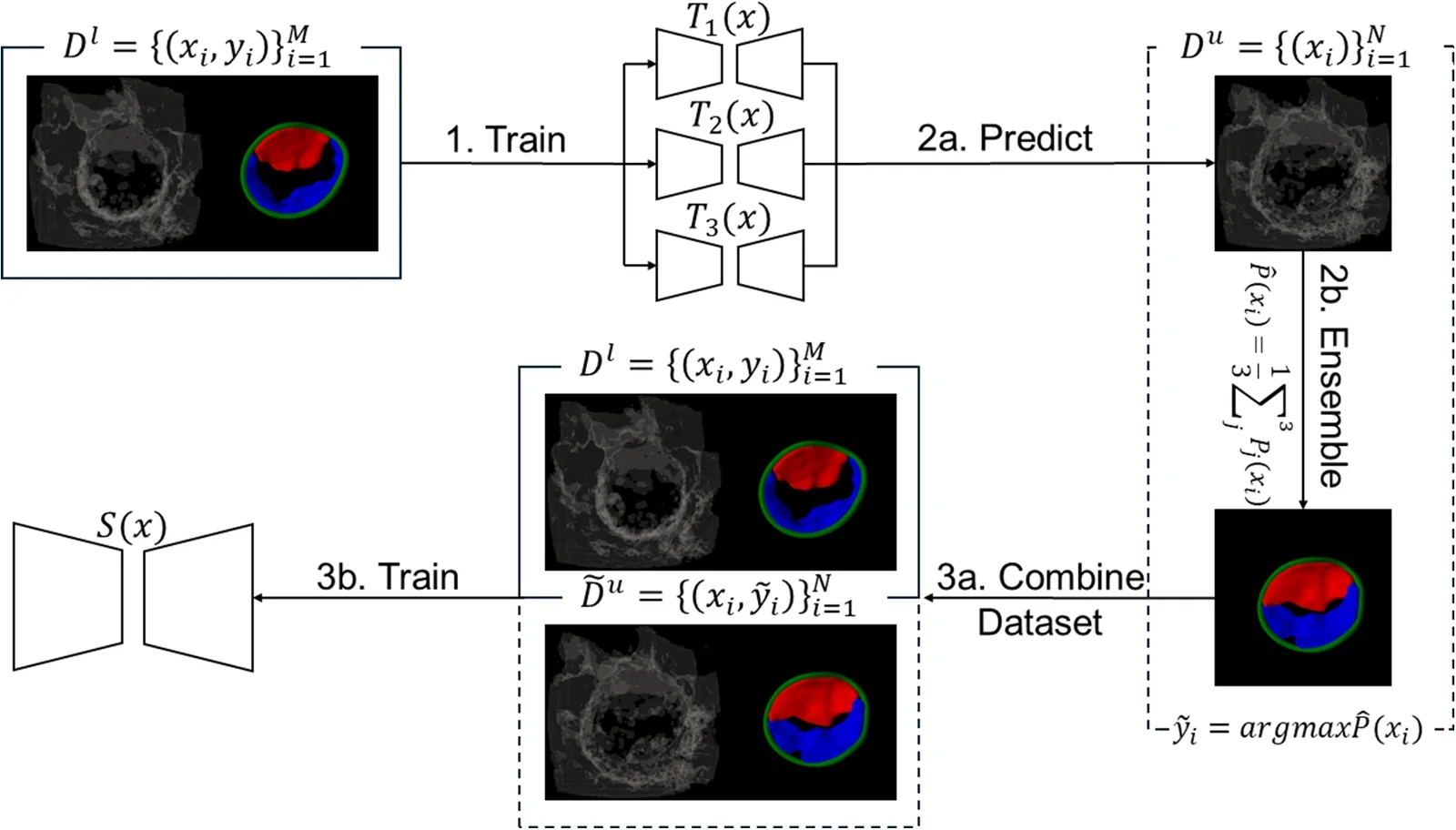
Overview of the proposed self-training strategy. Step 1, **Teacher Training**. $T_{1}$, $T_{2}$, and $T_{3}$ are trained on $D^{l}$, i.e., on 3D TEE images acquired at ES and ED. Step 2, **Pseudo Labelling**. (a) $T_{1}$, $T_{2}$, $T_{3}$ are used to make predictions on $D^{u}$, i.e., volumetric 3D TEE images acquired at intermediate systolic and diastolic frames of the cardiac cycle. Then, (b) pseudo-labels $\tilde{y}_{i}$ are generated by ensembling the predictions from the three models. Step 3, **Student Training**. (a) $D^{l}$ and $\tilde{D}^{u}=\{(x_{i}, \tilde{y}_{i})\}^{N}_{i=1}$ are combined, and (b) their combination is used to train the Student *S* model

For the formulation of the self-training approach, we leverage terminology specific to the Teacher-Student framework. In self-training, the Teacher model *T* is first trained on labeled data $D^{l}$ and then used to assign pseudo-labels to the unlabeled data point $x_{i} \in D^{u}$ [31]. The combination of the labeled and pseudo-labeled sets is used to train the Student model *S*. Previous formulations of self-training approaches may differ depending on the heuristics used to select the proxy-labeled examples to include in the labeled set. In this work, we propose to employ ensemble modeling to ensure reliable pseudo-labels. Specifically, we consider three different CNN architectures for image segmentation, which have been previously adopted for research studies [21, 33] or challenges [36] related to MV segmentation from 3D TEE images. These architectures are separately trained on $D^{l}$ using the same training scheme. The ensemble of these three models is then used to predict the pseudo-labels by averaging the probability maps from each model. Combining multiple networks into an ensemble typically improves results over using a single CNN [34]. Figure 2 shows a complete overview of the adopted self-training approach. Similarly to [32] and according to Fig. 2, the proposed self-training approach can be summarized in the following three steps:

1. **Teacher Training**: train three CNN models, $T_{1}$, $T_{2}$, $T_{3}$ on $D^{l}$.
2. **Pseudo Labelling**:
  - Use $T_{1}$, $T_{2}$, $T_{3}$ to make predictions on $D^{u}$.
  - Compute the average predicted probability map across the three models as $\hat{P}(x_{i}) = \frac{1}{3} \sum _{j}^{3}P_{j}(x_{i})$ with $x_{i} \in D^{u}$ and assign the pseudo-labels $\tilde{y}_{i} = \arg \max \hat{P}(x_{i})$ by taking the argmax of $\hat{P}(x_{i})$. Hence, in this formulation, the actual Teacher *T* consists of the ensemble of $T_{j}$, with j=1,2,3.
3. **Student Training**:
  - Generate the dataset to train the Student *S*. This is the combination of $D^{l}$ with $\tilde{D}^{u}$, where $\tilde{D}^{u}=\{(x_{i}, \tilde{y}_{i})\}^{N}_{i=1}$ is defined by combining each data $x_{i}\in {D}^{u}$ with the corresponding pseudo-label $\tilde{y}_{i}$.
  - Train a randomly initialized Student *S* on the combined $D^{l}$ and $\tilde{D}^{u}$.

In the described process, all datasets belong to the training set. Generalization performance will be evaluated on a held-out test set.

### Data augmentation on unlabeled images

To increase the robustness against less reliable pseudo-labels and ensure balanced performance across $D^{l}$ and $D^{u}$, strong data augmentation (SDA) is applied to unlabeled images during *S* training. This approach acts as a regularization technique, presenting a more challenging optimization target and preventing overfitting to less reliable pseudo-labels. Inspired by [32], Gaussian blurring and CutOut transforms [35] are included in the basic data augmentation routine. Gaussian blurring is an intensity-based transform that reduces image details while preserving edges by convolving the image with a Gaussian function. For this transform, sigma values are randomly selected between 0.5 and 1. CutOut is a spatial transform that randomly removes patches of a specific size from the images. For this transform, homogeneous patches with dimensions randomly selected between 2 and 20% of the image’s input dimensions are used. The application of both transforms on unlabeled images has been shown to be effective in self-training approaches, as demonstrated in [32].

To balance the impact of labeled and unlabeled data during *S* training, $D^{l}$ is oversampled to match the size of $D^{u}$. Specifically, each mini-batch is constructed to include an equal number of labeled and unlabeled images. This approach allows for balanced sampling of mini-batches from both datasets, ensuring that $D^{l}$ and $D^{u}$ equally contribute to each weight update during *S* training, thereby preventing bias towards the larger unlabeled dataset.

### Neural network architectures

Three architectures are considered for the Teacher models: Residual UNet, Multi-Decoder Residual UNet, and SegResNet. Residual UNet was successfully applied in [21] for MV 3D segmentation from 3D TEE images at ED. Multi-Decoder Residual UNet was proposed in our previous work [33] for efficient multi-structure MV 3D segmentation from 3D TEE images at ES. SegResNet won the MICCAI 2023 challenge on MV segmentation from 3D TEE [36]. These architectures share common beneficial features for MV segmentation from 3D TEE images but differ in complexity and in the number of trainable parameters.

#### Residual UNet

The Residual UNet is an encoder-decoder-based CNN architecture built upon the UNet framework [37]. It consists of five resolution levels, each one defined using residual units. In the encoder path, residual units include two convolutional blocks and an identity mapping. In the decoder path, the residual units include one convolutional block and an identity mapping. A transposed convolutional layer is used for upsampling. Each convolutional block includes a 3×3 convolutional layer with a stride of 2 in the encoding path, a batch normalization layer, and a parametric rectifying linear (PReLU) unit. This model has approximately 20 million trainable parameters.

#### Multi-Decoder Residual UNet

The Multi-Decoder Residual UNet is an extension of the Residual UNet with multiple decoder branches [33]. The backbone architecture is borrowed from the Residual UNet, but an equal number of convolutional blocks are used in the residual units of the encoder and decoder branches, resulting in a more symmetric structure. The number of decoder branches is extended to three, one for each detected class (mitral annulus, anterior leaflet, and posterior leaflet). This model has around 40 million trainable parameters.

#### SegResNet

The SegResNet is a UNet-based architecture with an asymmetrically larger encoder to extract image features and a smaller decoder to reconstruct the segmentation mask [38]. The encoder part uses ResNet blocks, which differ from previously described residual units by incorporating group normalization layers and ReLU units. Four stages of down-sampling are used, each stage has 1, 2, 2, and 4 ResNet blocks, respectively. In the decoder part, a single ResNet block per spatial level is used, and 3D bilinear upsampling restores the original input dimension. SegResNet is the lightest architecture considered, with around 18 million trainable parameters.

#### Teacher and Student architecture

A Residual UNet, Multi-Decoder Residual UNet, and SegResNet are used individually to construct $T_{1}$, $T_{2}$, and $T_{3}$, respectively. The prediction of these models is then ensembled to form the Teacher *T*. The ensemble approach exploits the strengths of each architecture by combining their predictions to generate more reliable and consistent pseudo-labels. For the Student *S*, SegResNet is chosen because of its lightweight architecture, which ensures shorter inference times.

### Training and implementation details

The Residual UNet, the Multi-decoder UNet, and the SegResNet are separately trained on $D^{l}$, using 70% of the images for training, 10% for validation, and 20% for testing. Data splitting is performed at the patient level to ensure that acquisitions from the same patient are not present in both training and evaluation sets. Images are resampled with a homogeneous spacing of 0.5 mm along each direction. Patches of $128 \times 128 \times 128$ voxels are randomly extracted from the input 3D image and passed to the model during the training phase. Data augmentation is performed *on-the-fly*, including intensity transforms such as intensity scaling and random Gaussian noise, as well as spatial transforms such as random rotation, random axis flip, and random elastic deformation. No transformation is applied during validation, whereas the largest connected component is extracted from the predicted segmentation during testing. Each model is trained over 500 epochs with a batch size of 2, using the Novograd optimizer [39] with a learning rate of 0.001. A weighted combination of Dice and Focal losses [40], with weights of 0.6 and 0.4, respectively, is minimized during the training phase. The saved model at the last epoch is used for inference on the test set.

After creating pseudo-labels $\tilde{y}_{i}$ in ${D}^{u}$, for the Student model, a new randomly initialized SegResNet architecture is trained on the combined set ${D}^{l}$ and ${D}^{u}$. The Student *S* is trained using the previously described data splitting and training scheme, with the addition of SDA on ${D}^{u}$. The basic data augmentation routine is augmented with random Gaussian blurring and random CutOut for the unlabeled dataset. All models were implemented using a PyTorch framework [41] and the Python-based medical imaging library MONAI [42]. Training and inference of the model were performed on an NVIDIA A4000 with 16 GB of memory.

## Evaluation

The test set consists of 16 4D TEE recordings. These are used to quantitatively evaluate if the proposed self-learning strategy fulfills two aims.

**Fig. 3:**
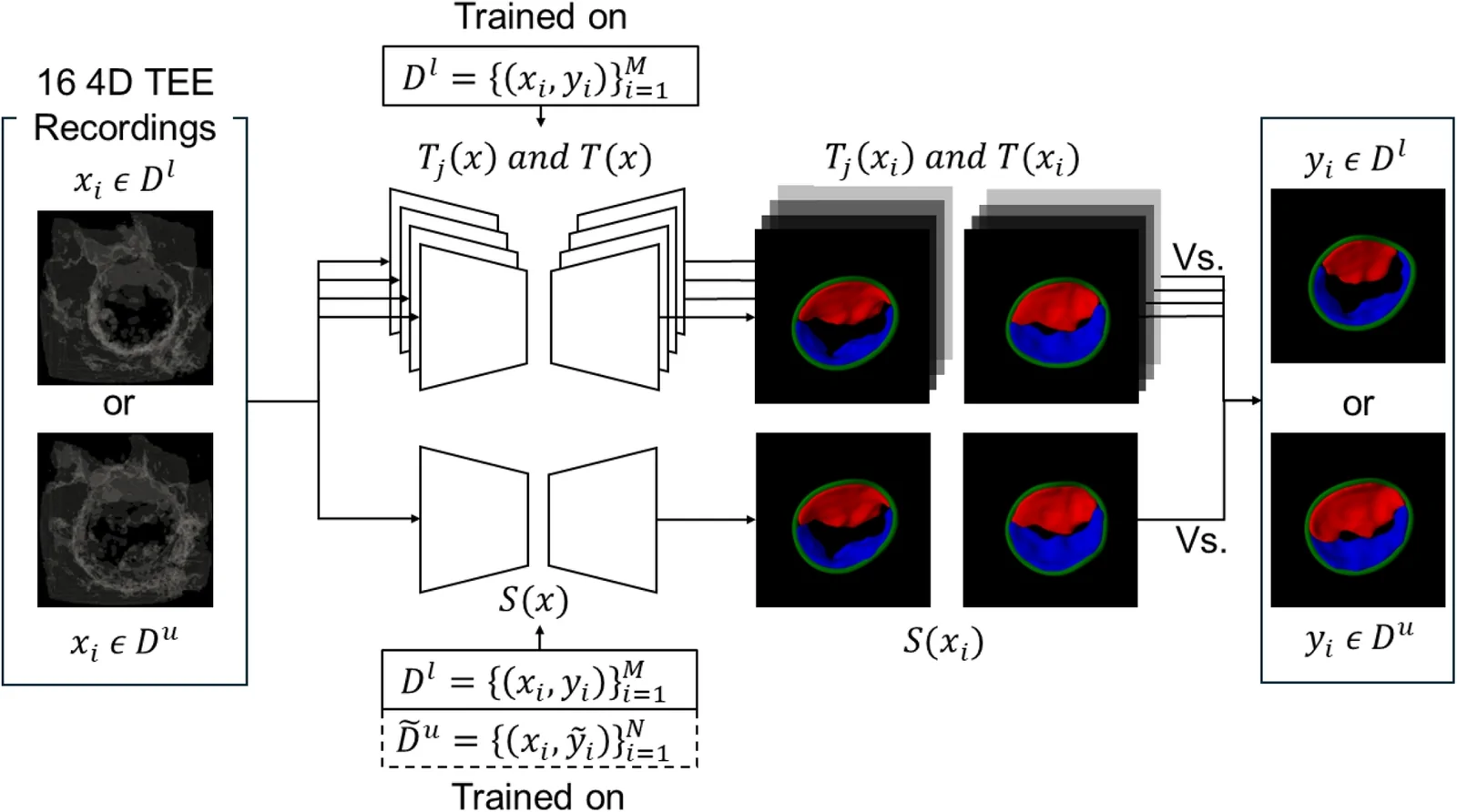
Schematic representation of the evaluation carried out on the 16 4D TEE recordings belonging to the test set. Inference of the individual Teachers $T_{j}$ and Ensemble *T* is performed separately on both ${D}^{l}$ and ${D}^{u}$, and compared against Student *S*. Note that Teachers are only trained on a limited labeled dataset ${D}^{l}$ whereas the Student *S* employs combined $D^{l}$ and $\tilde{D}^{u}$, leveraging a larger number of time frames

First, augmenting the training dataset for the Student *S* while maintaining high accuracy on the labeled data, demonstrating that pseudo-labels do not degrade performance. To this aim, each individual Teacher model $T_{j}$, the ensemble Teacher *T*, and the Student *S* are evaluated separately on $D^{l}$: the respective predictions $\hat{y}_{i}$ are compared vs. ground truth (GT) annotations $y_{i}$ by an expert operator for each $x_{i}\in {D}^{l}$ (Fig. 3). Standard segmentation metrics such as Dice score, average surface distance (ASD, in mm), and 95% Hausdorff distance (95%HD, in mm) are computed for the mitral leaflets. For the annulus, coordinate prediction metrics adopted in previous works [25, 26], referred to as curve-to-curve distance error (in mm) and in-plane error (in mm), are used. Curve-to-curve distance error represents the average shortest error between the predicted and the GT annulus 3D curve. In-plane error is the Euclidean distance between predicted and GT annulus points sampled from each rotating long-axis view around the left ventricle’s main axis in 3D TEE volumes. For more detailed definitions of the adopted metrics, we refer the reader to [26]. Metrics for the annulus are computed after the application of the reconstruction algorithm proposed in our previous work [33], which ensures consistent reconstruction of the whole annulus profile in case of under-segmented or noisy predictions. The tested hypothesis is that the predictions $\hat{y}_{i}$ yielded by the *S* model are at least accurate as the predictions $\hat{y}_{i}$ by any $T_{j}$ or by *T*.

Second, effectively improving generalization to unlabeled data and providing reliable and temporally consistent frame-by-frame MV segmentations over the cardiac cycle. To this aim, each individual Teacher model $T_{j}$, the ensemble Teacher *T*, and the Student *S* are evaluated separately on $D^{u}$ by comparing the respective predictions $\hat{y}_{i}$ vs. the GT segmentations $y_{i}$ for each $x_{i}\in {D}^{u}$ (Fig. 3). The metrics described in the previous paragraph are computed. The tested hypothesis is that the *S* model generalizes on frames that are not ES or ED better than any $T_{j}$ and than *T*, thanks to the training on a larger and more heterogeneous set that also includes $\tilde{D}_{u}$. Additionally, the overall temporal consistency (*TCS*) of the predicted segmentation is evaluated across each frame in a cardiac cycle of the available 4D TEE recordings in the test set. Similarly to [24], temporal consistency is evaluated by measuring a temporal consistency score, $TCS_t$, for MV prediction $\hat{y}_t$ at each frame *t* of the 4D TEE recording. $TCS_t$ is defined as:

1 $$\begin{aligned} TCS_t = \left\{ \begin{array}{ll} 1, &  \quad \text {if}~\hat{y}_t~\text {is consistent} \\ 0, &  \quad \text {otherwise} \end{array} \right. \end{aligned}$$

where $\hat{y}_t$ is considered consistent if the following two conditions are satisfied:

- The mitral annulus is segmented in at least 70% of the long-axis views identified in the 3D TEE volume.
- The relative change in volume ΔV of the predicted anterior and posterior leaflets between two consecutive frames is less than a specified threshold δ. This second condition leverages the incompressible behavior of leaflet tissue [44].

**Table 1:** Average segmentation performance across the ES and ED frames in the test set of $D^{l}$

	Teacher				Student	
Metric	$T_{1}$	$T_{2}$	$T_{3}$	*T*	*S* w/o SDA	*S* w/ SDA
Dice score	0.790±0.075	0.796±0.067	0.796±0.063	$\mathbf {0.806 \pm 0.085}$	0.799±0.051	$\mathbf {0.806 \pm 0.049}$
ASD (mm)	0.501±0.284	$\mathbf {0.423 \pm 0.137}$	0.446±0.168	0.425±0.191	0.449±0.135	0.442±0.139
95%HD (mm)	2.610±3.226	2.281±1.684	2.228±1.667	2.034±1.300	2.098±1.341	$\mathbf {2.019 \pm 1.258}$
Curve-to-curve (mm)	1.825±0.728	2.009±1.071	1.763±0.746	$\mathbf {1.686 \pm 1.120}$	1.920±0.903	1.865±0.728
In-plane (mm)	1.898±0.775	2.441±1.755	1.912±1.073	$\mathbf {1.818 \pm 3.252}$	2.231±1.571	2.109±1.073

Metrics include Dice score, ASD, 95%HD for the leaflets, and curve-to-curve error and in-plane error for the annulus. The Student *S* is evaluated with and without the application of SDA during model training. The best values for each metric across all models are highlighted in bold

**Fig. 4:**
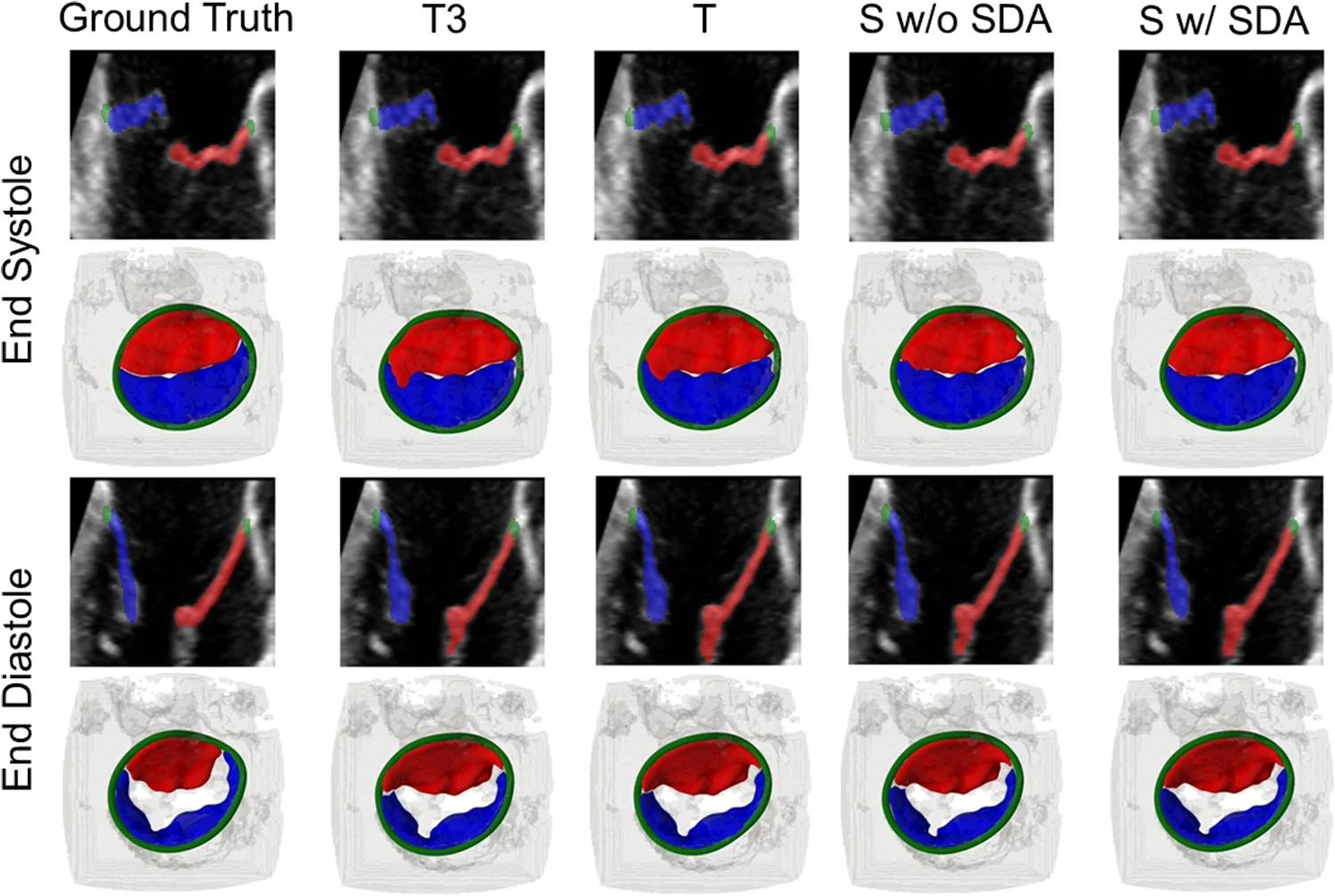
Segmentation examples from one 4D TEE recording in the test set. 2D views and 3D renderings of ES and ED frames from $D^{l}$ are shown along with the GT annotation and predictions from $T_{3}$, *T*, and *S*. Predictions for *S* are shown both with and without the use of SDA. The annulus is labeled in green, the anterior leaflet in red, and the posterior leaflet in blue

The first condition derives from the observation that the annulus reconstruction algorithm fails if the predicted segmentation of the annulus is missing from more than 30% of the long-axis views in the 3D TEE volume. The second condition verifies the physical consistency of the predicted 3D MV model by identifying the unacceptable volume changes in mitral leaflets predictions through time. The threshold δ is estimated as the average relative volume variation ΔV across the 4D TEE recordings in the test set, leveraging GT annotations of the frames sampled for constituting $D^{l}$ and $D^{u}$. δ is found to be 0.19 for the anterior leaflet and 0.15 for the posterior leaflet. *TCS* is the fraction of frames in a recording that pass the automatic consistency evaluation [24] and is computed for the dynamic MV predictions of Ensemble *T* and Student *S*, as well as the individual Teacher $T_{3}$, which shares the same SegResNet architecture with *S*. The segmentation consistency through time is further assessed by measuring the average absolute deviation (AAD) from the mean of the predicted anterior and posterior leaflet volumes across the cardiac cycle. This is given by

2 $$\begin{aligned} AAD_{mean} = \frac{1}{n_t} \sum _{t=1}^{n_t} |V_t - \bar{V}| \end{aligned}$$

where $n_t$ is the total number of frames in a cardiac cycle, $V_t$ is the leaflet volume estimation at frame *t*, and $\bar{V}$ is the mean volume estimation throughout the cardiac cycle. Lower variations in the volume estimation from the predicted segmentations consequently mean higher temporal consistency.

All metrics, including temporal consistency, are computed with and without the use of SDA to address the impact of this regularization technique on the performance of the *S* model.

## Results

### Performance on labeled dataset

Table 1 presents the average performance across the ES and ED frames in the test set of $D^{l}$. Overall, the Residual UNet ($T_{1}$), the Multi-Decoder Residual UNet ($T_{2}$), and the SegResNet ($T_{3}$), trained exclusively on $D^{l}$, achieve comparable segmentation quality for the leaflets, with Dice score values exceeding 0.79. Notably, the Multi-Decoder Residual UNet ensures lower ASD and 95%HD values (0.423±0.137 mm and 2.281±1.684 mm, respectively). For the annulus, the Residual UNet and SegResNet achieve curve-to-curve and in-plane errors less than 2 mm. As expected, the Ensemble *T* outperforms the individual architectures, achieving higher Dice score values (0.806±0.085), comparable ASD values (0.425±0.191 mm), and lower 95%HD values (2.034±1.300 mm) for the leaflets. The Ensemble *T* also ensures lower curve-to-curve error (1.686±1.120 mm) and in-plane error (1.818±3.252 mm) when identifying the annulus. The Student *S* shows comparable metrics to the Ensemble *T* and the single architectures in segmenting the MV at ES and ED frames. The use of SDA on $D^{u}$ slightly improves the performance on $D^{l}$ for the leaflets, yielding Dice score values of 0.806±0.049, ASD values of 0.442±0.139 mm, and 95%HD values of 2.019±1.258 mm, matching the performance of the Ensemble *T*. When labeling the annulus, although the use of SDA on $D^{u}$ results in some improvements, the Student *S* performs worse than the Ensemble *T*, with curve-to-curve and in-plane errors of 1.865±0.728 mm and 2.109±1.073, respectively. Figure 4 shows that the Student *S*, the Ensemble *T*, and the individual Teacher $T_{3}$ all yield MV segmentations that comparably match GT annotations when segmenting 3D TEE images at ES and ED. The Student *S* identifies more precisely the boundaries between anterior and posterior leaflets at ES.

**Table 2:** Evaluation performance across the three intermediate frames in the test set of unlabeled dataset $D^{u}$

	Teacher				Student	
Metric	$T_{1}$	$T_{2}$	$T_{3}$	*T*	*S* w/o SDA	*S* w/ SDA
Dice score	0.799±0.058	0.732±0.133	0.802±0.048	0.809±0.049	0.810±0.054	$\mathbf {0.815 \pm 0.040}$
ASD (mm)	0.413±0.143	0.383±0.097	0.406±0.156	0.382±0.101	0.469±0.548	$\mathbf {0.373 \pm 0.091}$
95%HD (mm)	2.061±1.719	5.168±6.153	2.135±1.590	1.995±1.682	2.059±2.352	$\mathbf {1.719 \pm 1.043}$
Curve-to-curve (mm)	1.406±0.440	3.593±3.677	1.444±0.479	$\mathbf {1.357 \pm 0.432}$	1.751±1.601	1.391±1.321
In-plane (mm)	1.632±0.999	5.389±7.237	1.691±1.581	$\mathbf {1.420 \pm 0.645}$	2.995±4.325	2.428±4.084

For the Student *S*, performance metrics are reported with and without the application of SDA during model training. The best values for each metric across all models are highlighted in bold

**Fig. 5:**
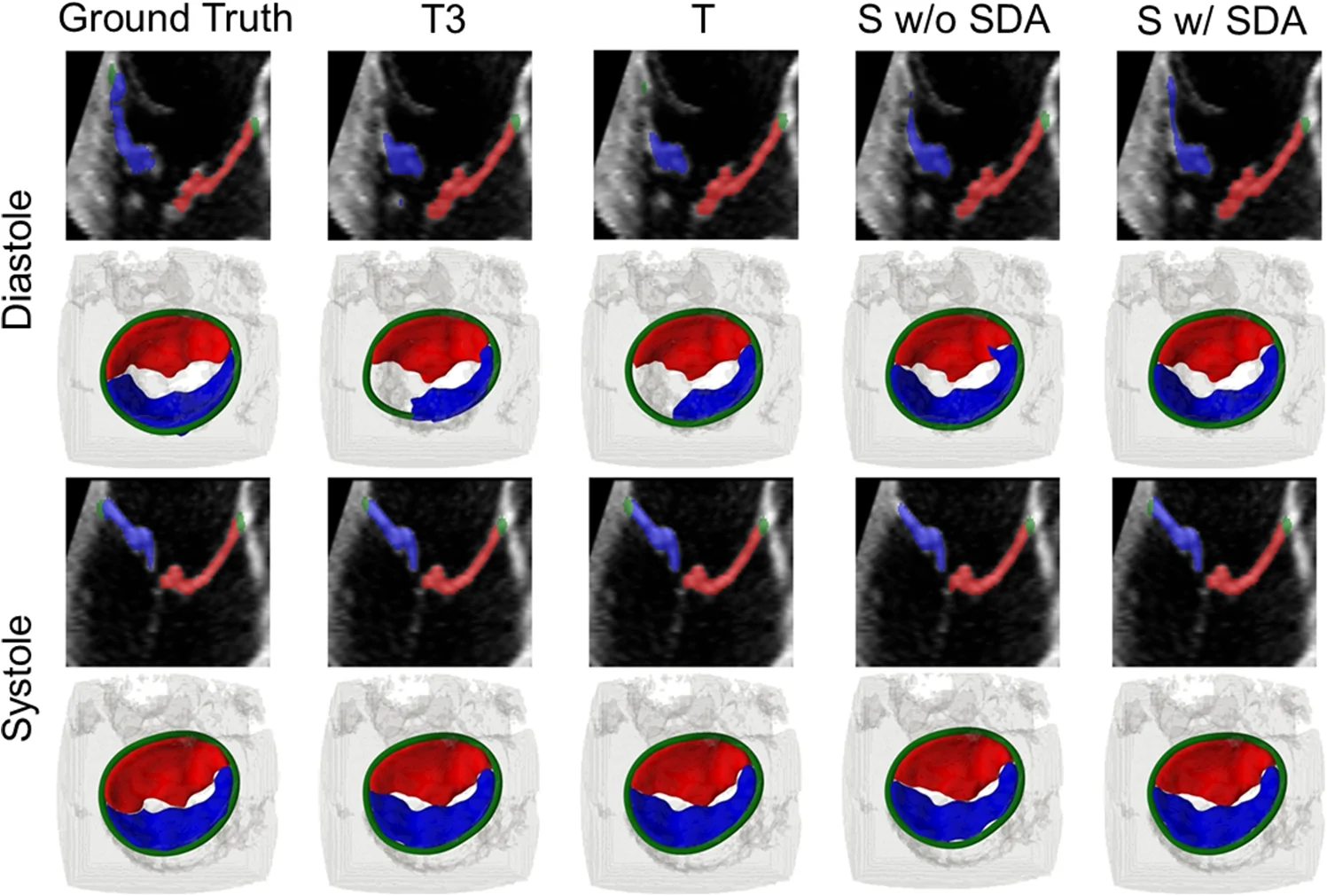
Segmentation examples from one 4D TEE recording in the test set. 2D views and 3D renderings of generic diastole and systole frames from $D^{u}$ are shown along with the GT annotation and predictions from $T_{3}$, *T*, and *S*. Predictions by the Student *S* with and without the use of SDA are shown. The annulus is labeled in green, the anterior leaflet in red, and the posterior leaflet in blue

**Fig. 6:**
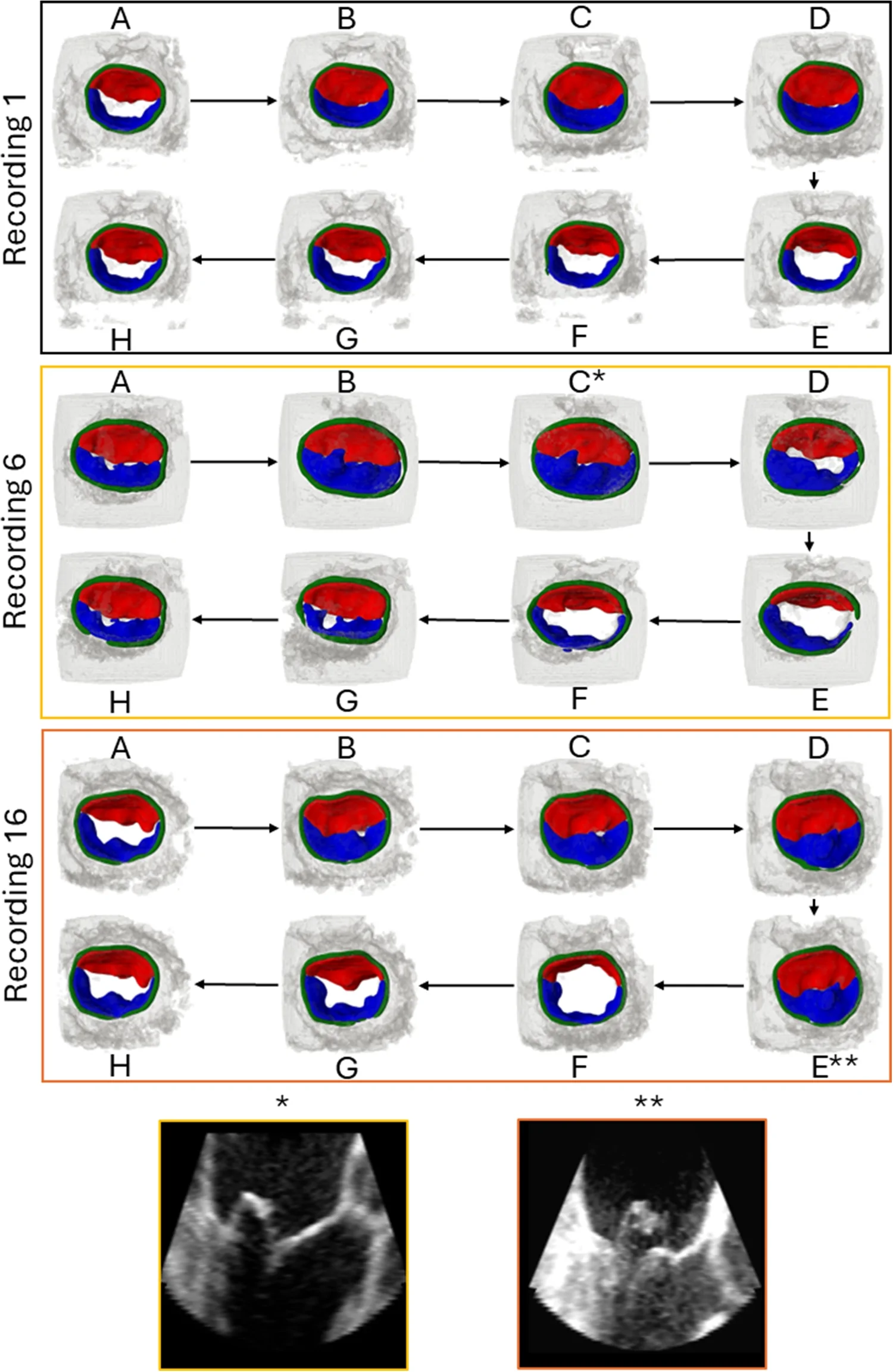
Automatic frame-by-frame segmentation from three randomly selected 4D TEE recordings. Each differently colored rectangle represents a distinct 4D TEE recording, with black arrows and letters indicating the chronological sequence. At the bottom, two long-axis views taken from systolic frame C of Recording 6 and frame E of Recording 16 highlight the prolapsing posterior leaflet profile correctly segmented by Student *S*. Color code: annulus is green, anterior leaflet is red, and posterior leaflet is blue

### Performance on unlabeled dataset

Table 2 reports the average performance across the three intermediate frames in the test set of $D^{u}$. Remarkably, the Multi-Decoder Residual UNet ($T_{2}$) shows degraded performance when evaluated on the unseen intermediate frames of $D^{u}$, with lower Dice score values and higher distance metrics for both the leaflets and the annulus as compared to the performance achieved on $D^{l}$. In contrast, the Residual UNet ($T_{1}$) and the SegResNet ($T_{3}$) demonstrate higher generalization capability on unseen frames as compared to $T_{2}$, achieving Dice score values exceeding 0.79, ASD values lower than 0.5 mm, and 95%HD around 2 mm for the leaflets. For the annulus, $T_{1}$ and $T_{3}$ achieve curve-to-curve and in-plane errors lower than 1.7 mm. The Ensemble *T* consistently outperforms the individual architectures and generalizes well to the unseen intermediate frames of $D^{u}$. It achieves Dice score values of 0.809±0.049, ASD values of 0.382±0.101 mm, and 95%HD values of 1.995±1.682 mm for the leaflets, along with low distance values for the annulus. The performance metrics achieved by the Ensemble *T* on $D^{u}$ underscore the consistency provided in the generation of the pseudo-labels for the training of the Student *S*. As compared to the Ensemble *T*, the Student *S* provides comparable performance metrics in terms of Dice score (0.810±0.054) for the leaflets, but slightly lower values in terms of distances, particularly for the annulus. Notably, the Student *S* benefits from the use of SDA on $D^{u}$ during training, consistently improving the Dice score values up to 0.815±0.040 and lowering the ASD and 95%HD values to 0.372±0.091 mm and 1.719±1.043 mm, respectively, surpassing the Ensemble *T*. For the annulus, all the metrics improve with SDA, although curve-to-curve and in-plane errors remain worse than those obtained by the Ensemble *T*. Figure 5 shows an illustrative comparison where the Student *S* outperforms the Ensemble *T* and the individual Teacher $T_{3}$ in segmenting the MV in a generic diastolic frame pertaining to $D^{u}$. The use of SDA on $D^{u}$ further improves the segmentation, making it more consistent with the GT annotations. However, in systole the better performance of the Student *S* and the improvement introduced by the additional use of SDA are less evident.

**Fig. 7:**
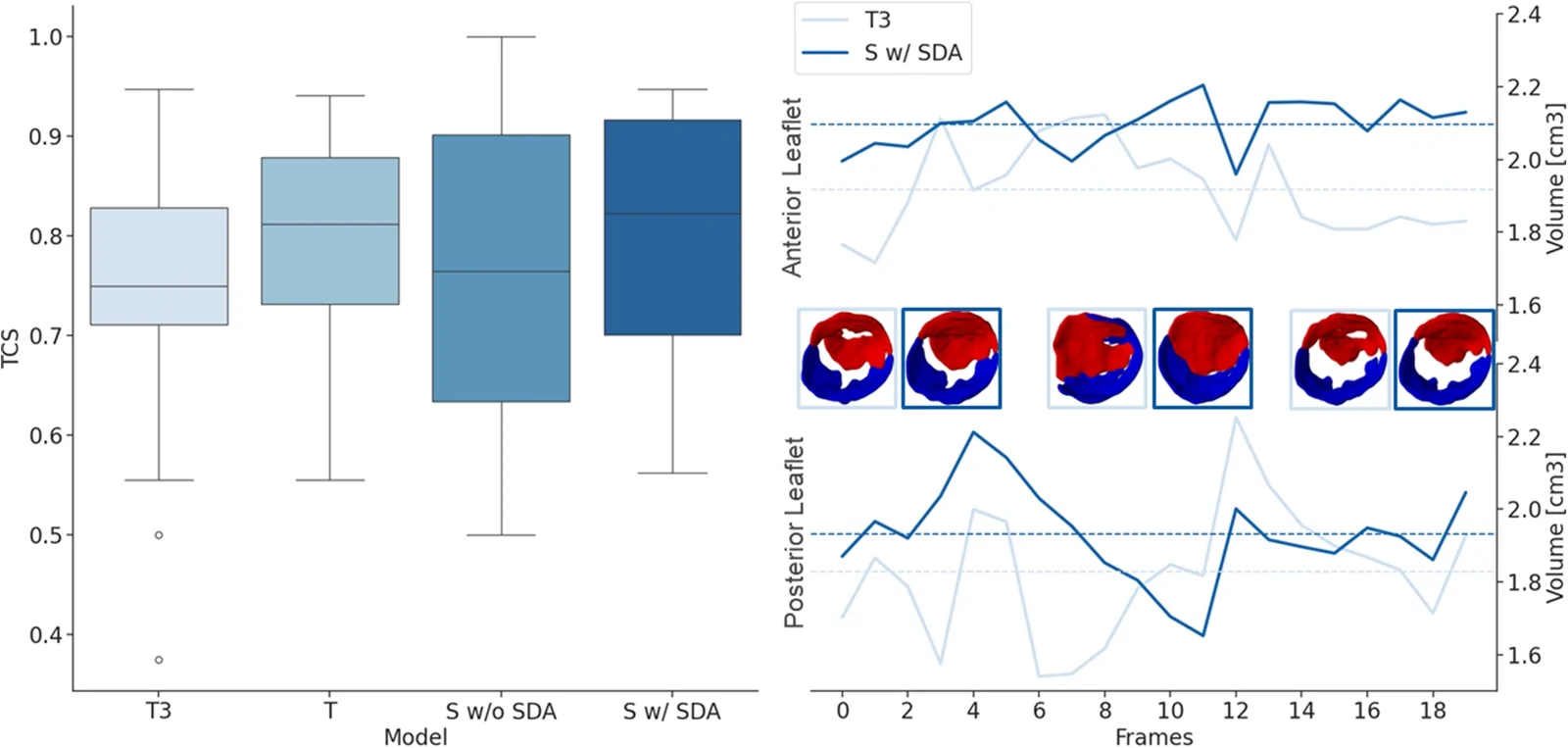
Boxplot of temporal consistency results for individual Teacher $T_{3}$, the Ensemble *T*, and the Student *S* with and without the applications of SDA (left side). Illustrative example of predicted anterior and posterior leaflet volume predictions from a 4D TEE recording provided by $T_{3}$ and *S* with SDA. Snapshots of MV segmentation from the frames are also shown (right side)

### Frame-by-frame segmentation

Figure 6 shows the effect of the proposed self-training strategy to achieve MV 4D segmentation from 4D TEE recordings. The Student *S* provides coherent estimation of the MV dynamics and morphology throughout the cardiac cycle in different 4D TEE recordings. Notably, the reconstruction of MV leaflets yielded by the Student *S* is accurate enough to allow for clearly assessing leaflet billowing or prolapse and coaptation gaps, when present, in the systolic frames, i.e., when the MV is closed (Fig. 6, Recording 6 and Recording 16). The predicted annulus presents discontinuities in some phases of MV dynamics, which can be resolved by applying the reconstruction algorithm proposed in [33]. Figure 7 compares TCS for the individual Teacher $T_{3}$, the Ensemble *T*, and the Student *S*. For clarity, only the individual Teacher $T_{3}$ is considered in this analysis because it shares the same SegResNet architecture with the Student *S* and provides better overall segmentations than the other individual Teachers on $D^{l}$ and $D^{u}$. The figure also exemplifies the variability in leaflets volume prediction for $T_{3}$ and *S* (Fig. 7, right). The Student *S* provides higher TCS values but with high variability across the test set (Fig. 7). The introduction of SDA on $D^{u}$ leads to overall better temporal consistency with median value of 0.823, exceeding both the individual Teacher $T_{3}$ (0.750, p=0.25,p>0.05) and the Ensemble *T* (0.812, p=0.83,p>0.05). The predicted leaflet volumes in Fig. 7 (right panel), shows that the Student *S* provides more temporally regular segmentations that are closer to the mean value than $T_{3}$, especially for the anterior leaflet, achieving a lower AAD of 0.054 $cm^{3}$ compared to 0.108 $cm^{3}$ of $T_{3}$. This is qualitatively observed in the snapshots representing the MV predictions from the two models at three consecutive time frames (Fig. 7, right). Table 3 reports AAD of the predicted leaflets volume across each frame of 4D TEE recordings in the test set. On average, the Student *S* with SDA and the Ensemble *T* achieve the lowest average absolute deviation for the anterior leaflets (0.122±0.041 $cm^{3}$ and 0.122±0.044 $cm^{3}$, respectively). However, for the posterior leaflets, the use of SDA for training *S* degrades leaflet consistency throughout the cardiac cycle, resulting in an average AAD of 0.192±0.081 $cm^{3}$, matching the performance of the individual Teacher $T_{3}$.

**Table 3:** Average absolute deviation of predicted anterior leaflet (AL) and posterior leaflet (PL) volume estimation over the cardiac cycle

Average Absolute Deviation	$T_{3}$	*T*	*S* w/o SDA	*S* w/ SDA
AL Volume (cm$^3$)	0.148±0.039	0.122±0.044	0.135±0.060	$\mathbf {0.122 \pm 0.041}$
PL Volume (cm$^3$)	0.192±0.081	0.175±0.073	$\mathbf {0.165 \pm 0.093}$	0.192±0.088

Mean and standard deviation are computed across each 4D TEE recording of the test set and reported for model $T_{3}$, the Ensemble *T* and the Student model *S* with and without the applications of SDA

The lowest average absolute deviations of predicted anterior leaflet and posterior leaflet volume estimation are highlighted in bold

Overall, the Student *S* provides consistent temporal MV segmentations, requiring an average inference time of 0.182±0.038 s per 4D TEE recording and 0.010±0.001 s per single frame. Although, *S* achieves comparable or slightly better MV temporal consistency than the Ensemble *T*, which requires 19.888±5.478 s to infer 4D MV segmentation, and 1.169±0.272 s per single frame.

## Discussion

### Novelty and added value of the proposed method

This study addresses a significant gap in automatic and standardized 4D TEE MV segmentation by introducing a novel application of a semi-supervised training strategy that leverages pseudo-labeling. The proposed approach reduces the reliance on manual annotations, while achieving high segmentation accuracy across the cardiac cycle, enhancing clinical applicability.

Although an exact comparison cannot be made, given the difference in the used dataset, the individual Teachers ($T_{1}$, $T_{2}$, $T_{3}$) report on $D^{l}$ results comparable to previous studies which focus only on MV segmentation at the ES frame [33], at the ED frame [21, 36] or at both frames [23] and report Dice score values ranging between 0.81 and 0.877, ASD values between 0.32 and 0. 92 mm, and 95% HD values between 1.86 and 1.99 mm. Similarly, the lowest curve-to-curve and in-plane errors obtained by $T_{1}$ and $T_{3}$ for the annulus are comparable to previous works reporting curve-to-curve and in-plane errors equal to 2.04 mm and 1.94 mm [25], or 1.96 mm and 1.82 mm [26]. However, the Ensemble *T* consistently outperforms the individual Teachers $T_{j}$, ensuring higher Dice score (0.81) for the leaflets and lower curve-to-curve and in-plane errors (1.67 mm and 1.81 mm, respectively). This demonstrates the advantage of ensemble methods in providing more accurate and robust predictions by averaging out biases [34]. The use of the pseudo-labels, produced by the Ensemble *T* for $D^{u}$, in training the Student *S* does not negatively impact the performance of the Student *S* on $D^{l}$; instead, it ensures comparable performance with respect to the Ensemble *T*, especially with SDA applied during training. This indicates that the Ensemble *T* generates reliable pseudo-labels, and SDA mitigates potential errors in pseudo-labeling. Quantitative evaluation of the negative impact of label noise in deep neural network training is achievable through various techniques [47, 48]. However, these methods require a subset of data free of label noise or at least knowledge of the noise level in labels, information that is often prohibitively costly to obtain. Stochastic co-teaching has recently been proposed to address this limitation. It involves sequentially training two models with identical architectures on the same data but with different instance selections. In this framework, each model selects training instances for the other based on GT posterior probabilities and a stochastically chosen threshold [49]. This method eliminates the need for assumptions regarding the expected level of label noise. Nonetheless, we believe that an in-depth analysis of label noise impact would deviate from the main scope of this work, especially given the favorable results on the labelled dataset ensured through pseudo labelling using ensemble modelling. Previous studies [50, 51] have applied an ensemble method in self-training strategies to mitigate the risk of incorrect pseudo-label, which may cause severe error propagation referred to as confirmation bias. In these works, each member of the ensemble consisted of the same network, but was trained with different stochastic input augmentation. Here, we chose to exploit three different CNN architectures for ensemble modeling. By combining their strengths and compensating for their weakness we effectively address error propagation. The proposed self-training strategy is then effective in augmenting the training dataset for the Student *S*, enhancing segmentation performance.

The evaluation on $D^{u}$ highlights the generalization ability over multiple frames of the cardiac cycle, i.e., the capability to correctly segment MV substructures in open, closed, or intermediate configurations of the MV. The Multi-Decoder Residual UNet ($T_2$) exhibits degraded performance on unseen time frames, likely due to the higher complexity of its architecture that makes it more prone to overfitting. In contrast, $T_1$ and $T_3$ demonstrate good generalization abilities, overall maintaining the same performance showed on $D^{l}$. As expected, the Ensemble *T* again outperforms the individual Teachers $T_{j}$ for both mitral leaflets and annulus segmentation in terms of Dice score, ASD, and 95%HD values. Notably, the Student *S* overall equals the segmentation performance of the Ensemble *T* but improves the mitral leaflets Dice score values, suggesting higher generalization capabilities and more reliable MV segmentation than *T*. SDA further improves the results for leaflets segmentation, guaranteeing the lowest ASD and 95%HD values, and the best Dice score. The results achieved on $D^{u}$ demonstrate the effectiveness of the proposed self-training strategy in improving generalization performance as compared to the Teachers. We believe that this improvement is due not only to the additional training examples provided for the Student *S* but also to the choice of training a randomly initialized model on a combined labeled and pseudo-labeled dataset from the start. This approach helps the Student *S* learning more generalized patterns by introducing variability at the beginning of the training process. Randomly initialized weights have been found to be effective in improving generalization capability and mitigating the performance lag typically experienced in two-phase learning regimes using pre-trained models [52, 53]. Therefore, this choice acts as a regularization technique which, together with the use of SDA [32], contributes to learning robust features from pseudo-labeled data.

The proposed strategy achieves unprecedented automatic 4D MV segmentation, with the Student *S* providing consistent segmentations across the cardiac cycle, accurately detecting anatomical features of regurgitant MV, such as prolapses (Fig. 6). Indeed, the Student *S* provides better *TCS*, when SDA is applied, in 4D MV segmentation as compared to the individual Teacher $T_{3}$, which shares the same SegResNet architecture with Student *S*, and to the ensemble *T*. Although these differences are not statistically significant (p>0.05), probably due to the limited sample size in the test set (16 4D TEE recordings), the Student *S* exhibits fewer fluctuations in predicted leaflet volumes across frames (Fig. 7), confirmed by a low mean AAD for anterior leaflet volume and for posterior leaflet volume. These results confirm the effectiveness of the proposed method in improving the generalization performance. The Student *S* is better equipped to capture the rapid changes in MV configurations though each frame of a 4D TEE recording regardless of the temporal resolution considered. This leads to a more reliable MV dynamic segmentation. However, the application of SDA contributes to reducing the AAD for the anterior leaflet but not for the posterior leaflet. Of note, the MV posterior leaflet exhibits particularly high shape variability in 4D TEE recordings which is challenging to address in automatic segmentation methods. Indeed, the posterior leaflet often opens toward the direction of the ultrasound waves, which determines loss of information due to signal dropout [46]. Consequently, this aspect may influence the analysis of temporal consistency, which is based on the assumption of constant leaflet volume during the cardiac cycle, because leaflet tissue is incompressible [44], and any permissible variation in volume of the segmentation mask must be associated with limitations of echocardiography, such as signal dropout.

To the best of our knowledge, this is the first work which presents and evaluates an automatic method for 4D MV with focus on regurgitant MVs. Unlike a previous work that only qualitatively demonstrated 4D MV segmentation [23], our work extensively and quantitatively evaluates the proposed strategy, comparing predicted results with expert manual annotations and introducing criteria for temporal consistency evaluation. Moreover, the multi-class segmentation task enriches the value of the proposed strategy by providing separate segmentations of the mitral annulus, anterior, and posterior leaflets. Finally, as compared to the Ensemble *T* the Student *S* is not only comparably reliable, but also almost 100 times faster, on average, in segmenting the 4D TEE data, as detailed in Sect. 4.3

### Clinically relevant fallouts

The accuracy, automation, and time-efficiency of the proposed Student *S*, as well as its capability to provide frame-by-frame segmentations of MV substructures, make it a potentially useful tool to support diagnosis and procedural planning.

Indeed, two main types of MR exist, which are characterized by completely different pathophysiological mechanisms and require different management [55]: organic, which is related to primary alterations of MV substructures, and functional, which is secondary to the adverse remodeling of the left ventricle. The most common form of organic MR is degenerative, which encompasses a broad range of alterations in MV tissues.

The patients selected in the present study were all affected by degenerative MR. When managing this type of patients, the detailed quantification of MV morphology and dynamics over the cardiac cycle is key to correctly classifying MR etiology within the spectrum of degenerative conditions, and hence to selecting the most suitable MV repair procedure [54]. The approach herein proposed would allow for such a detailed quantification based on 4D TEE: it allows not only identify leaflet billowing and regurgitation gaps at ES, when the MV would be perfectly sealed if healthy, but also to assess MV dynamics thanks to reliable frame-by-frame segmentation. Also, the full automation of the segmentation and reconstruction process would make the quantification operator-independent and fully repeatable, while decreasing the workload of sonographers and improving clinical workflow efficiency.

The time required by the Student *S* to process a single cardiac cycle in a 4D TEE recording make it suitable for intra-procedural use. In particular, it could be useful in transcatheter MV repair, which is performed in beating hearts and requires the continuous monitoring, through 4D TEE, of MV position and motion in the intracardiac space [56].

Additionally, the automatic reconstruction analysis of MV dynamics from 4D TEE could be used to improve numerical models for the quantification of MV biomechanics, which are proposed in the literature as a means to better tailor surgical or transcatheter treatments to the patient [57, 58]. The data yielded by the Student *S* could be used to feed those models, by providing the unloaded MV geometry at ED and annular motion to be imposed as boundary condition or to validate them, by providing the GT time-dependent configuration of the MV.

### Limitations and future works

The choice of Teacher architectures affects the generation of pseudo-labels, requiring models with good generalization abilities. Exploring other architectures, such as Attention UNet, which proved robust against image artifacts [24], could improve pseudo-label accuracy and robustness against signal dropout-related issues. Additionally, the proposed method is validated on a relatively small, homogeneous dataset of MR patients. Future work should include larger, more heterogeneous datasets, e.g., including MR with other etiologies, to confirm robustness. Furthermore, incorporating MV temporal consistency as a regularization technique in the training process could enhance dynamic segmentation. Finally, integrating the segmentation framework with real-time TEE imaging systems could facilitate its use in intraoperative settings, providing surgeons with real-time feedback during MV examinations or repair procedures.

## Conclusion

We proposed a semi-supervised segmentation strategy that significantly advances 4D TEE MV segmentation by reducing the need for extensive manual annotations while achieving high segmentation accuracy and temporal consistency. This work formulates a self-training strategy with pseudo-labeling, based on the Teacher-Student framework, to address the lack of training data for dynamic MV segmentation in 4D TEE. The generation of pseudo-labels using an ensemble of three different CNN architectures and the application of a peculiar SDA on the pseudo-labeled dataset were effective in increasing the generalization performance for temporal segmentation of the Student model. Our approach has strong potential for clinical applications.
